# Senescence profiling and biomarker identification in cell product based on adipose tissue–derived mesenchymal stromal cells

**DOI:** 10.1093/stcltm/szag011

**Published:** 2026-03-07

**Authors:** Ellen Mønsted Johansen, Cecilie Hoeeg, Rebekka Harary Søndergaard, Lisbeth Drozd Højgaard, Laura Lykke Lethager, Stine Bangsgaard, Jens Kastrup, Tu Hu, Thomas Litman, Morten Juhl Nørgaard

**Affiliations:** Cardiology Stem Cell Centre, The Heart Centre, University Hospital of Copenhagen Rigshospitalet, Copenhagen 2100, Denmark; Cardiology Stem Cell Centre, The Heart Centre, University Hospital of Copenhagen Rigshospitalet, Copenhagen 2100, Denmark; Cluster for Molecular Imaging, University of Copenhagen, Copenhagen 2200, Denmark; Cardiology Stem Cell Centre, The Heart Centre, University Hospital of Copenhagen Rigshospitalet, Copenhagen 2100, Denmark; CelltoCure Aps, Birkerød 3460, Denmark; Cardiology Stem Cell Centre, The Heart Centre, University Hospital of Copenhagen Rigshospitalet, Copenhagen 2100, Denmark; Cardiology Stem Cell Centre, The Heart Centre, University Hospital of Copenhagen Rigshospitalet, Copenhagen 2100, Denmark; Cardiology Stem Cell Centre, The Heart Centre, University Hospital of Copenhagen Rigshospitalet, Copenhagen 2100, Denmark; Cardiology Stem Cell Centre, The Heart Centre, University Hospital of Copenhagen Rigshospitalet, Copenhagen 2100, Denmark; CelltoCure Aps, Birkerød 3460, Denmark; True Signal, Copenhagen 2300, Denmark; Department of Immunology and Microbiology, University of Copenhagen, Copenhagen 2200, Denmark; Cardiology Stem Cell Centre, The Heart Centre, University Hospital of Copenhagen Rigshospitalet, Copenhagen 2100, Denmark

**Keywords:** senescence, adipose tissue–derived mesenchymal stromal cells, biomarkers, gene expression, quality control, cell therapy

## Abstract

**Background/aim:** Adipose tissue–derived mesenchymal stromal cells (ASC) are used in advanced therapy medicinal products due to their regenerative and immunomodulatory properties. Increasing the number of dosages derived from each donor product is essential to reduce variability and improve scalability of cell therapy. However, extended in vitro expansion may induce cellular senescence, potentially compromising therapeutic efficacy. This study aimed to assess the remaining proliferative potential of a cryopreserved ASC product and identify robust transcriptomic ­biomarkers of senescence.

**Methods:** ASC from five donors were cultured until replicative senescence or passage 10. Morphology, growth kinetics, and confluence were monitored. Bulk RNA sequencing was performed on samples from passage 1, 3, 6, and final passage. Principal component analysis, differential expression, gene set variation analysis, and variance partitioning were used to characterize transcriptional changes and identify biomarkers.

**Results:** ASC maintained stable proliferation and morphology for at least three passages post-thaw. Major transcriptional shifts occurred between passage 3 and later passages. Senescence-associated gene enrichment increased progressively, with donor-specific variation evident at intermediate passages. Forty biomarkers (20 upregulated, 20 downregulated) were identified with expression changes primarily attributable to passage rather than donor identity.

**Conclusion:** Cryopreserved ASC retain substantial proliferative capacity post-thaw. Senescence develops gradually and is detectable through consistent transcriptomic changes. These findings relate to proliferative and senescence-associated molecular changes and do not directly assess therapeutic efficacy. The identified biomarkers provide a foundation for developing senescence-focused quality control assays to support safe and effective ASC-based therapies.

Significance statement This study identifies promising biomarker candidates for developing a quality control assay to detect cellular senescence and evaluates the remaining replicative potential of a GMP-approved investigational medicinal product. These findings contribute to improving the safety and consistency of cell-based therapies by enabling detection of senescence-related changes.

## Graphical abstract

**Graphical Abstract:**
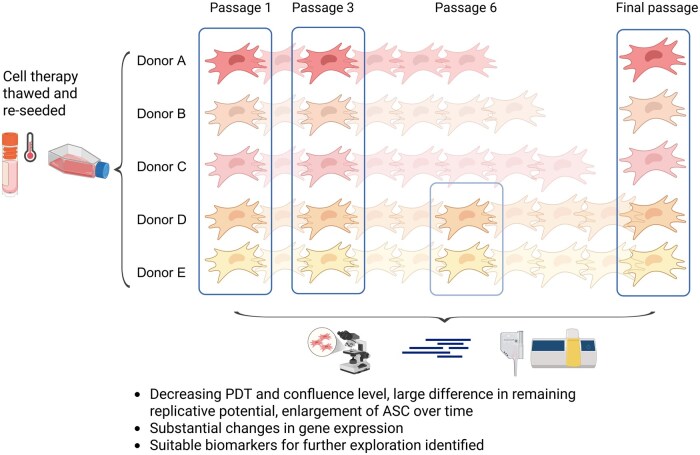

## Introduction

Advanced therapy based on mesenchymal stromal cells (MSC) originating from, for example, adipose tissue (ASC) shows promise in treatment of various conditions such as nonischemic heart failure and graft-versus-host disease.^1^^,^^2^ Advanced therapies are highly regulated, and manufacturers must comply with Good Manufacturing Practise standards, including strict quality control, to ensure patient safety and a consistent quality of the product.^3^ Cardiology Stem Cell Centre (CSCC) has developed a cryopreserved, allogeneic ASC-based cell product expanded in hollow-fiber bioreactors and tested it clinically in more than 300 patients.^1^^,^^4–8^ The CSCC cell product undergoes quality control, including sterility and immunophenotype, in accordance with good manufacturing practice.^9^ Functional assays, based on previous research, are used to quantify the angiogenic and immunomodulatory properties.^10–13^

Adipose tissue-derived mesenchymal stromal cells undergo numerous population doublings, and cells from a single donor are expandable to billions.^9^ Currently, the cell therapy developed by CSCC is cultured for two passages, representing a minimal risk of cellular senescence.^14^ Expanding each batch of cell therapy to include more population doublings (PDs) could increase the number of doses obtained from each adipose tissue donor, thereby reducing production costs. Since each treatment dose originates from a single donor, fewer donors would be needed to treat the same number of patients, which could in turn reduce product variability. Such an approach could contribute to make the cell product feasible as a standard treatment. However, prolonged cell proliferation will eventually lead to cellular senescence, potentially impairing the immunomodulatory properties and therapeutic function of the cell therapy.^15–17^ Senescent cells are characterized by mitochondrial dysfunction, increased oxidative stress, and accumulation of cellular damage. They also secrete a range of pro-inflammatory cytokines, chemokines, growth factors, and matrix-degrading enzymes—collectively referred to as the senescence-associated secretory phenotype (SASP).^18^ Cell therapy unintentionally based on senescent ASC will deviate from the intended characteristics (target product profile) of the cell therapy, potentially resulting in reduced efficacy or even causing adverse effects in patients. Therefore, extended proliferation makes thorough quality control focused on cellular senescence, complemented by functional assays, a prerequisite.

While senescence has been associated with impaired cellular function, the present study focuses on proliferative capacity and senescence-associated molecular changes rather than direct assessment of therapeutic efficacy.

The aim of this study was therefore to investigate the remaining replicative potential of the cell product, its temporal morphology, and growth kinetics. Additionally, biomarker candidates for cellular senescence in ASC were identified through transcriptomic analysis. Suitable biomarker candidates were required to be sufficiently expressed for reliable detection and show large, significant differential expression between first passage after thawing (passage 1) and the final passage. Differences in gene expression levels between passage 1 and final passages needed to account for the majority of variance, and finally, the biomarkers had to be biologically relevant, eg, associated with processes, such as proliferation, aging, or characteristics linked to SASP.

Candidate biomarkers may provide further insight into senescence-related changes within ASC populations and could potentially be used in the development of a future quality control assay for the release of cell products for clinical use.

## Materials and methods

### Cell culture

Recruitment of human donors was completed with informed consent in connection with clinical trials approved by the regional scientific ethical committee, Capital Region of Denmark. The cell material originating from these human donors used for this study is classified as a medicinal product and can be used for research with the purpose of quality control of the specific medicinal product without further ethical approval.

Cultured ASC were derived from the CSCC cell product^9^ originating from five adipose tissue donors. During production, the ASC underwent two passages in a hollow-fiber bioreactor and were cryopreserved following each passage. Population doublings and passages during manufacturing were excluded from analysis to focus on remaining proliferative potential.

On the same day, all five cell products were simultaneously thawed in a 37 °C water bath and resuspended in pre-warmed culture medium (CM), αMEM without nucleosides (Thermo Fisher/Gibco), supplemented with penicillin and streptomycin (100 U/mL and 100 μg/mL, Thermo Fisher/Gibco) and 5% heparin-free human platelet lysate (Sexton Biotechnologies/BioLife Solutions).

Cells were seeded at a density of 10^3^ ASC/cm^2^ in surface-treated culture flasks (Nunc), starting in T75 flasks and scaling to T175/T500 to increase yield. Cultures were incubated at 37 °C, 5% CO_2_, with CM completely changed twice/week. Cells were harvested after 7 days for the first three passages and before reaching confluence in the later passages.

During harvest, ASC were washed with PBS (Thermo Fisher/Gibco) and detached using 1× TrypLE Select (Thermo Fisher/Gibco) at 37 °C (<10 min). The ASC were counted on a NucleoCounter^®^ NC-100 (Chemometec) and passaged.

The ASC were passaged until either senescent, defined as cessation of population growth where fewer ASC were harvested than seeded, or until passage 10. Parameters evaluating growth kinetics were calculated as follows:


(1) $$\mathrm{Population}\;\mathrm{Doublings}\;(PD)=\;\frac{\ln\left(\mathrm{harvested}\;\mathrm{viable}\;\mathrm{ASC}\right)-\ln(\mathrm{seeded}\;\mathrm{viable}\;\mathrm{ASC})}{\ln(2)}$$

(2) $$\mathrm{Cumulative}\;\mathrm{Population}\;\mathrm{Doublings}\;(\mathrm{cPD})=PD(p_{1})+PD(p_{2})+\cdots +PD(p_{n})$$

where PD represents the population doublings of each passage, from passage 1 (*p*_1_) to final passage *n* (*p_n_*).


(3) $$\mathrm{Population}\;\mathrm{Doubling}\;\mathrm{Time}\;(\mathrm{PDT})=\frac{\Delta \mathrm{days}}{PD}$$

where Δdays is the time interval (in days) between seeding and harvest, and *PD* is the number of population doublings that occurred during that time interval.

### Cryopreservation

After passaging, remaining ASC were centrifuged at 300*g* for 5 min and resuspended in CryoStor (CS10) (Sexton Biotechnologies/Bio Life Solutions) at concentrations of 5 × 10^5^-1 × 10^6^ ASC/mL. In later passages, lower yields resulted in lower concentration for cryopreservation. Samples of 1 mL were transferred to 1.8 mL cryotubes (Nunc), placed in a pre-cooled CoolCell container (BioCision), frozen at −80 °C overnight (−1 °C/min), and stored in liquid nitrogen. Samples were cryopreserved after passage 1, passage 3, and final passages for bulk RNA sequencing. Samples from donors D and E, that reached passage 10, were also cryopreserved after passage 6.

### Image analysis and confluency evaluation

Morphology and growth kinetics were assessed at every passage. For that purpose, three images were captured from random center positions, 2 to 3 times per week following CM change, using an EVOS™ XL Core microscope (Invitrogen) equipped with a 10× LWD PH objective.

Confluence levels were quantified using a custom automated batch macro in FIJI (ImageJ)^19^ that utilized the PHANTAST plugin.^20^ The pipeline included Median blur filtering and exclusion of particles smaller than 500 pixels^2^ to generate binary masks and calculate confluence.

### Bulk RNA transcriptomics

Cryopreserved ASC samples were shipped on dry ice for RNA extraction and sequencing. The samples represented five donors at passage 1, five at passage 3, two at passage 6, and five at their final passage. The aim was to investigate longitudinal changes in gene expression across passages.

For three of the five donors, proliferative capacity declined substantially at later passages. To prioritize continuation of the cultures toward replicative senescence, all available ASC were used for further passaging. Consequently, insufficient cell material was available for additional cryopreservation and downstream analyses at passage 6.

#### Library preparation and sequencing

RNA extraction, library preparation, and sequencing were performed as follows: total RNA was extracted using Trizol, and mRNA was enriched using oligo(dT)-attached magnetic beads. The enriched mRNA was fragmented and reverse transcribed to synthesize complementary DNA (cDNA). The resulting cDNA was end-repaired, ligated with adapters, and amplified by PCR. Following denaturation, single-stranded circular DNA libraries were generated and quality-checked. Sequencing was conducted on the DNBSEQ platform using 100 base pair paired-end reads of short RNA sequences. Raw reads were processed using SOAPnuke^21^ to remove adapter contamination, reads with “*N*” content <0.1%, and low-quality reads, defined as those with more than 20% of bases having a quality value <15.

#### Alignment and mapping reads

The original locations of the short reads were determined by aligning them to a reference genome and read counts from individual genes were quantified.

File integrity was verified using md5sum. Alignment was carried out using Salmon (v1.10.3), with reference genome Ensembl Homo sapiens GRCh38 (v113), using the nf-core/rnaseq pipeline (v3.17.0). Only protein-coding genes were retained for downstream analysis.

All analyses were conducted in Rstudio (v4.4.0)^22^^,^^23^ within a secure, ISO/IEC 27001:2013 certified HPC environment, and documented in reproducible markdown files.

#### Dimension reduction and unsupervised analysis

Unsupervised data analysis was performed to investigate sample grouping and expression patterns. Raw count data were normalized to library size and variance stabilization was applied using the rlog function from DESeq2 R package.^24^ Genes with low expression (median counts ≤1 across all samples) were excluded from further analysis. The top 500 variable genes were selected for principal component analysis (PCA) and heatmap. The PCA was conducted using the base R function prcomp, and heatmap was generated using the R package ComplexHeatmap.^25^

#### Differential expression and Venn diagram

Differential expression analysis was performed across three contrasts: Passage 1 to passage 3, passage 1 to final passage, and passage 3 to final passage. Passage 6 was not included due to limited replicates (*n* = 2).

Gene expression was modeled using passage as the explanatory variable (∼passage) in the DESeq2 R package. Log2 fold change shrinkage was applied to all three contrasts using the lfcShrink function.^26^

Genes were filtered according to Log 2-Fold Change (LFC) > 1 (upregulated) or LFC < −1 (downregulated), adjusted *P*-value <.05, and baseMean >150. A Venn diagram was constructed with the ggvenn R package^27^ to visualize the distribution of unique and shared genes across the contrasts.

#### Variance partitioning and biomarker selection

Variance partition analysis was performed to identify robust biomarker candidates and only samples from passage 1 and the final passage were included. The dataset was filtered to include only genes represented in the Venn diagrams, for the contrast passage 1 vs. final passage, both unique and shared genes. Variance partitioning was conducted using the variancePartition R package^28^ to quantify the fraction of variance that was attributable to passage and donor for each selected gene. Prior to analysis, counts were log2-transformed using the edgeR R package.^29^

The final biomarker candidates were selected based on the variance attributable to passage (>0.80). From these, the top 40 upregulated and top 40 downregulated based on LFC were identified, and finally, the top 20 statistically significant genes from each group, based on adjusted *P*-values, were kept.

#### Gene set variance analysis and gene set enrichment analysis

The relative level of senescence in each of the 17 samples was assessed using the gene set variation analysis (GSVA) package in R^30^ applying the gene set SAUL_SEN_MAYO containing 125 genes upregulated in cells exhibiting SASP.^18^ The gene set has been shown to be consistently upregulated across multiple tissues^31^ and, when validated against several cell types, it was shown to outperform other senescence-related gene sets.^32^

Additionally, a Gene Set Enrichment Analysis (GSEA) was performed using Gene Ontology (GO) terms to identify enriched biological processes (BP).^33^ This was done with the gseGO function from the clusterProfiler package in R,^34^ focusing on the contrast passage 1 vs. final passage.

All genes with median counts ≥1 across all samples were included in both the GSEA and GSVA analyses, ranked by log2foldchange.

### Data visualization

Figure 2 was prepared in inkscape (version 1.0.1). Remaining figures were prepared in R studio.

## Results

### Cell culture until replicative senescence

**Figure 1.**
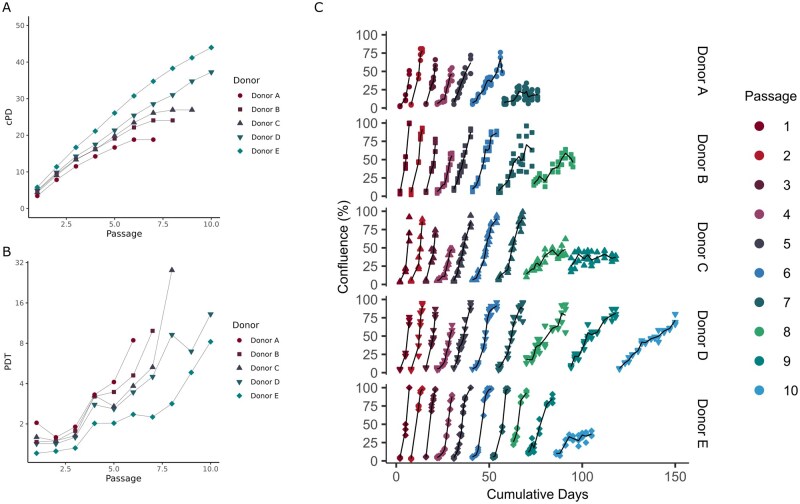
ASC from five ASC donors (A-E) cultured for 7-10 passages following thawing of final cell product. (A) Cumulative population doublings (cPD) over passages and (B) population doubling time (PDT, log 2 scale) for each passage (no PDT data for final passage of donor A-C since fewer ASC were harvested than seeded). (C) Confluence over time (days). Passages are shown over time (days) and represented with different colors. Fluctuations in confluence represents each passage. Donors are represented in separate rows in the figure.

ASC from all five donors (A to E) were able to proliferate when cultured following thawing; however, a large variation in cPD and PDT was observed (see Figure 1). This was illustrated by the extremes: donor A, which proliferated for 77 days and achieved 18.8 cPDs over 7 passages, whereas cells from donor E proliferated for 107 days and reached 44 cPDs over 10 passages (see Figure 1A). The ASC cultures were considered senescent when fewer ASCs were harvested, than initially seeded. The culture was discontinued at this point, and the remaining ASC were cryopreserved. Cultures from donors A-C was stopped prior to passage 10; therefore, we have no data for PDT in the final passages as the number of harvested ASC was lower than the seeded ASC (Figure 1B). The PDT in passage 10 of donors D and E were comparable to the PDT of the last recorded PDT of donors A to C.

The cultures were passaged once per week the first three passages with a mean cPD of 13.8 ± 1.9 (SD). During this time, PDT remained stable (Figure 1B). Thereafter, PDT gradually increased, and ASC were harvested before reaching confluence in up to 10 passages. Figure 1C illustrates that confluence increases over time within each passage and that this increase progressively slows down with each subsequent passage. Eventually, the cell populations were no longer able to reach full confluence, entering a plateau where the ASC ceased to proliferate.

### Morphology

**Figure 2.**
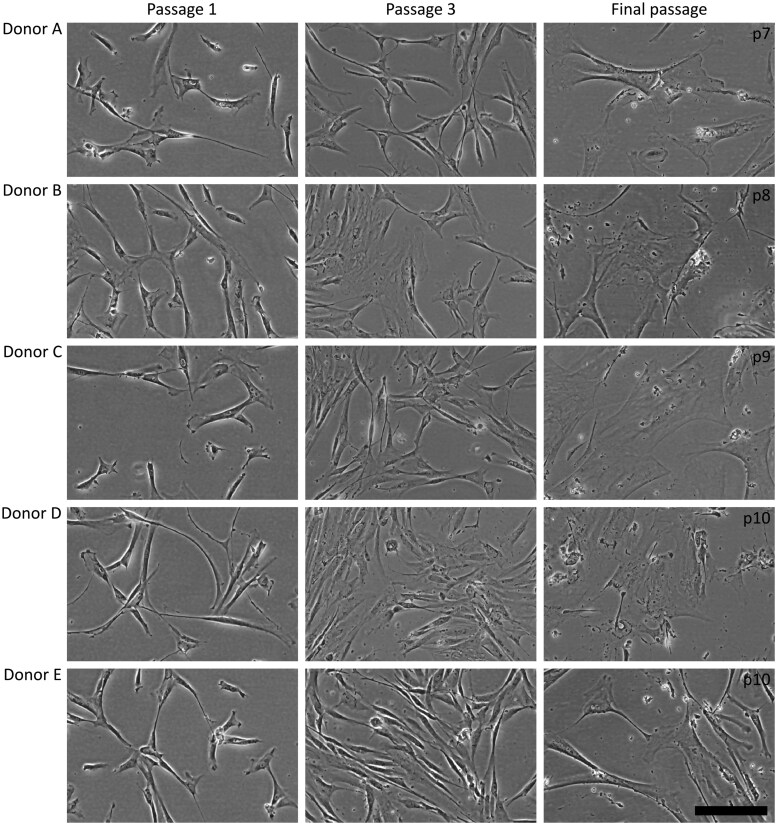
Representative photos of the five donors. Passage 1 after thawing (p1), Passage 3 (p3), and the final passages (p7-p10), scale 200 µm.

The cell morphology changed over successive passages. In the first passage after thawing, the ASC population from all donors appeared small and spindle-shaped (Figure 2). By passage 3, the cultures had undergone a substantial number of population doublings but still exhibited a morphology comparable to passage 1, with the exception of a few enlarged ASC, for example, donor B. In final passage (passage 7-passage 10), the ASC were clearly enlarged.

### Analyses of gene expression

RNA from ASC passages 1, 3, 6, and the final passage was isolated and sequenced to investigate transcriptomic changes across successive passages.

#### Principal component analysis and heatmap

**Figure 3.**
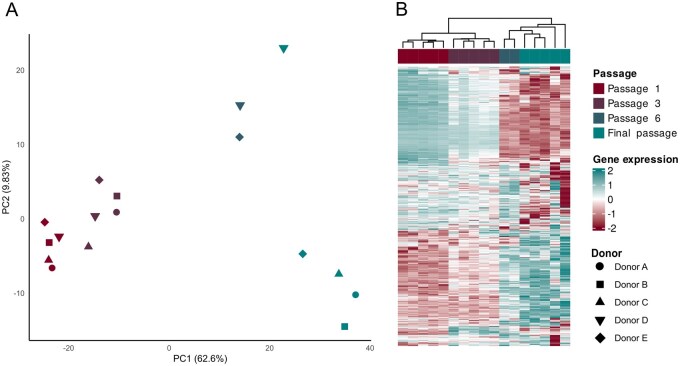
(A) Principal component analysis (PCA) plot, where colors show passage, and symbols show donors and (B) heatmap showing unsupervised hierarchical clustering of samples along with passage. The 500 most varying genes are used in both PCA and heatmaps.

Samples clustered with passage in the PCA plot (Figure 3A) and a clear separation of passage 1/passage 3 from passage 6/final passages, primarily along PC1. Some separation of passage 1 and passage 3 along PC1, and to a lesser extent PC2, was observed. The major shift occurred, however, between passage 3 and passage 6/final passages, indicating major transcriptomic changes during these passages.

These observations were supported by the heatmap (Figure 3B), which showed that the Euclidian distance between passages 1 and passage 3 ASC was smaller than the distance between passages 3 and passage 6/final passages of ASC.

#### Differential expression

**Figure 4.**
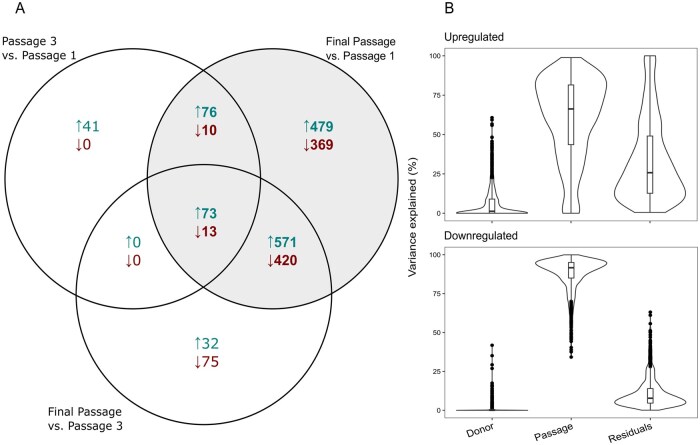
(A) Venn diagram showing unique and shared upregulated (maroon) and downregulated genes (teal) in the three different contrasts from the DESeq2 analysis. Only genes with Log2FoldChange >1 or <−1, adjusted *P*-value <.05 and Base Mean >150 are represented. Grey area depicts the genes used for in the downstream analysis. (B) Violin plots illustrate the distribution of variance attributable to Donor, Passage, and Residuals of Upregulated genes, LFC >1, adjusted *P*-value <.05, and BaseMean >150 and Downregulated genes, LFC <−1, adjusted *P*-value <.05, and BaseMean.

We observed substantial changes in gene expression across passages. The differentially expressed genes were filtered (absolute LFC > 1, baseMean > 150, adj. *P* < .05). After filtering, we found 213 genes in passage 3 vs. passage 1, 1184 genes in final passage vs. passage 3, and 2011 genes in final passage vs. passage 1. There were both unique and shared genes across passages. These are depicted in Figure 4A. The majority of unique genes were found in the comparison between final passage vs passage 1. In contrast, fewer genes were differentially expressed between passage 3 and passage 1, supporting the observations from the PCA and heatmap.

Samples from passage 6 were included for exploratory visualization only and were excluded from formal differential expression analyses due to limited sample size (*n* = 2).

#### Variance partition

Variance in gene expression attributable to passage and donor was calculated between donors in passage 1 and in the final passage (Figure 4B) to assess sources of variance. Passage accounted for the largest proportion of variance in both upregulated and more prominently, in downregulated genes. In contrast, donor identity contributed minimally to overall variability across gene expression.

Residual variance, representing the portion of variability, not explained by donor or passage, differed between upregulated and downregulated genes (Figure 4B). Downregulated genes exhibited consistently low residual variance across genes, suggesting a more uniform response across donors, whereas upregulated genes displayed a broader distribution of residual variance.

#### Selection of biomarkers

**Figure 5.**
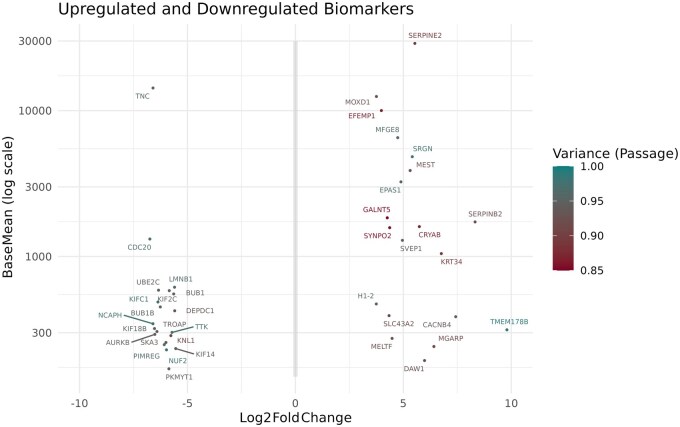
Upregulated (Log2Foldchange > 1) and downregulated genes (Log2Foldchange < −1) as suitable biomarkers. *X*-axis shows log2foldchange from −10 to 10, and *Y*-axis shows BaseMean from 150 to 30 000. Color indicates how much of the variance in gene expression is attributable to Passage.

Twenty upregulated and 20 downregulated potential biomarker genes were selected based on their variance attributable to passage, LFC, and adjusted *P*-value, all shown in Figure 5 and Table 1. The upregulated genes exhibited a broad distribution across all three parameters, as illustrated in Figure 5 and Table 1: BaseMean (average count number) (193—28 886), LFC (3.7 to 9.8), and variance attributable to passage (0.85-0.99).

**Table 1.** Upregulated and downregulated genes showing Log2FoldChange (LFC), baseMean, variance across passage (Passage), and adjusted *P*-value (*P*adj).

Upregulated genes					Downregulated genes				
SYMBOL	LFC	Passage	BaseMean	*P*adj.	SYMBOL	LFC	Passage	BaseMean	*P*adj.
**CACNB4**	7.4	0.94	386.00	3.9e-38	**AURKB**	−6.5	0.95	292.00	1.2e-92
**CRYAB**	5.7	0.87	1602.00	1.6e-28	**BUB1**	−5.6	0.95	553.00	9.7e-93
**DAW1**	6.0	0.93	193.00	3.8e-41	**BUB1B**	−6.3	0.95	451.00	6.8e-96
**EFEMP1**	4.0	0.87	10008.00	8.3e-29	**CDC20**	−6.7	0.97	1317.00	1.0e-118
**EPAS1**	4.9	0.96	3248.00	1.8e-77	**DEPDC1**	−5.6	0.93	424.00	5.1e-76
**GALNT5**	4.3	0.85	1841.00	3.3e-30	**KIF14**	−5.5	0.95	234.00	3.2e-72
**H1-2**	3.7	0.96	473.00	1.3e-50	**KIF18B**	−6.5	0.95	320.00	3.6e-63
**KRT34**	6.8	0.90	1046.00	7.5e-39	**KIF2C**	−5.8	0.95	583.00	5.3e-88
**MELTF**	4.5	0.93	274.00	3.0e-40	**KIFC1**	−6.4	0.98	487.00	1.1e-122
**MEST**	5.3	0.93	3884.00	2.8e-46	**KNL1**	−5.8	0.91	287.00	1.8e-73
**MFGE8**	4.7	0.96	6523.00	2.4e-86	**LMNB1**	−5.6	0.96	616.00	6.5e-94
**MGARP**	6.4	0.91	241.00	2.3e-38	**NCAPH**	−6.6	0.98	346.00	8.3e-101
**MOXD1**	3.8	0.93	12496.00	7.4e-31	**NUF2**	−6.0	0.98	229.00	1.7e-66
**SERPINB2**	8.3	0.92	1725.00	1.6e-29	**PIMREG**	−6.1	0.96	249.00	1.5e-95
**SERPINE2**	5.5	0.90	28887.00	6.3e-30	**PKMYT1**	−5.9	0.95	170.00	1.1e-59
**SLC43A2**	4.3	0.92	393.00	9.7e-34	**SKA3**	−6.0	0.93	257.00	3.7e-85
**SRGN**	5.4	0.98	4831.00	1.6e-98	**TNC**	−6.6	0.96	14292.00	5.6e-75
**SVEP1**	5.0	0.95	1290.00	5.8e-39	**TROAP**	−6.4	0.94	306.00	1.1e-80
**SYNPO2**	4.4	0.86	1575.00	2.3e-33	**TTK**	−5.7	0.97	302.00	3.6e-90
**TMEM178B**	9.8	0.99	314.00	1.9e-36	**UBE2C**	−6.3	0.95	587.00	5.3e-74

The downregulated genes on the other hand showed a more uniform distribution, with BaseMean values ranging from 169 to 14 292, LFC from −5.5 to −6.7, and variance attributable to passage between 0.91 and 0.98.

It is worth noting that expression of classical MSC-associated markers, including NT5E (CD73), THY1 (CD90), and ENG (CD105), all remained highly expressed across passages, and HLA and PTPRC(CD45) were not detected and therefore absent in Figure S1—see online supplementary material for a color version.

#### Gene set enrichment analyses

**Figure 6.**
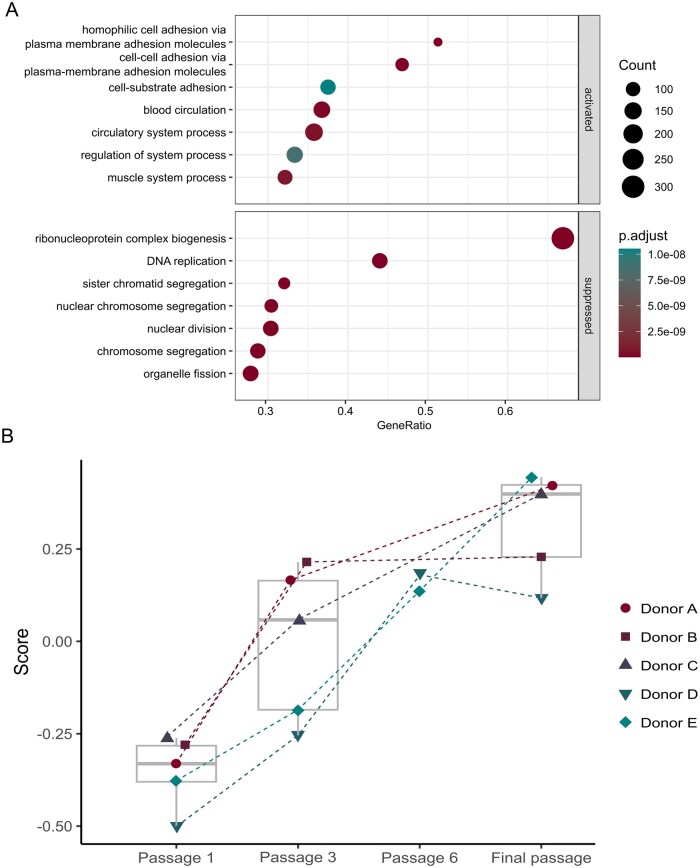
(A) Gene Set Enrichment Analysis of Gene Ontology, biological process. Count = number of genes in the gene list, which is represented in the gene set. Gene ratio is counts/number of genes in the gene list. (B) The GSVA score (*y*-axis) for each of the investigated passages (*x*-axis). Legend shows shape for each donor (Donors A-E) and boxplots show median and interquartile range.

Gene Ontology (GO) enrichment analysis was conducted to identify biological processes that differed between ASC in their final passage and passage 1. The activated gene sets (Figure 6A, top panel) suggest that final passage ASC exhibit increased adhesion properties and enhance processes associated with tissue remodeling and support for vascular or muscular systems, which are consistent with Figure 2, where enlarged ASC in final passage adhere to a larger surface.

In contrast, the suppressed gene sets (Figure 6A, bottom panel) were related to pathways associated with cell proliferation and mitosis, consistent with the decrease in proliferation, as shown in Figure 1.

#### Gene set variance analysis

The SenMayo gene set^18^ was used in a GSVA to assess the enrichment of senescence-associated genes (Figure 6B). ASC from passage 1 had the lowest score, while those from the final passage exhibited the highest. Samples from passage 3 and passage 6 displayed intermediate scores. The boxplot of passage 3 samples is the widest, as a result of higher variance in GSVA scores in this passage. Interestingly, ASC from donor B scored as high in passage 3 as in its final passage (passage 8).

## Discussion

Manufacturers of ASC-based advanced therapies rely on the ability of ASC to expand to numbers relevant for clinical dosages and at the same time production costs must be reduced for cell therapies to represent a realistic option for standard care. However, ASC are heterogenous and dynamic cell populations, which develop when cultured,^35–37^ and with prolonged in vitro cultivation, ASC will eventually be rendered senescent. Manufacturers must find a balance where costs are reduced, while maintaining safety and efficacy of ASC-based therapies. Therefore, an increase in population doublings must be accompanied by the implementation of a quality control assay evaluating senescence.

Importantly, this study was not designed to directly assess repair efficacy or therapeutic potency. Although functional assays are routinely applied to the ASC product, the present analyses are limited to proliferative behavior, morphology, and senescence-associated transcriptional changes.

We demonstrated that the investigational ASC product, for use in clinical trials, retained a considerable replicative potential. While no universal parameters for senescence are defined, our conclusion is based on the three key observations. (1) Proliferation and morphology: Upon thawing, ASCs maintained a stable population doubling time for at least three passages. During this period, they predominantly exhibited a small, spindle-shaped morphology, similar to that seen in passage 1. Additionally, proliferation continued until at least passage 7. (2) Transcriptomic stability: Unsupervised gene expression analysis supported these findings. PCA revealed tight clustering of passage 1 ASC, with passage 3 forming an adjacent cluster, indicating only minor transcriptional changes. In contrast, the clusters of samples from passage 6 ASC (only available for donors D and E) and final passages clearly separated from passages 1 and 3. Additionally, this pattern was confirmed by heatmap analysis, which showed a lower Euclidean distance between passages 1 and 3 compared to the distance between passage 3 and the later passages. (3) Differential gene expression: The number of unique and shared differentially expressed genes was markedly higher in comparisons between the final passage and passage 1 or passage 3, than between passages 1 and 3. Many DEGs were shared across contrasts, suggesting a gradual progression. Overall, the data strongly suggests that the most significant changes emerge between passage 3 and the onset of senescence. Despite extensive changes in transcriptomic profile, the expression of classical MSC markers remained highly expressed also in final passages, supporting the need to supplement immunophenotyping with more targeted assays.

Our GO enrichment analysis revealed a clear shift in final passage ASC from proliferation-associated processes toward increased expression of genes involved in adhesion, wound healing, and vascular- and muscle-related pathways. This expression pattern is consistent with previous reports of MSC senescence, in which cell cycle-associated genes, such as those involved in mitosis, DNA replication, and chromosome segregation, are downregulated during extended culture.^38–40^ Moreover, senescent MSC have been shown to upregulate genes related to extracellular matrix and adhesion, which may contribute to impaired migration and altered regenerative function.^41–43^ These consistent transcriptional changes highlight the progressive nature of replicative senescence and suggest that late-passage ASC may transition toward a static, matrix-remodelling phenotype at the expense of proliferative potential.

Senescence in a cell population is a gradual process, where the fraction of senescent cells increases with replicative exhaustion.^44^ To assess this, GSVA was performed using the SAUL_SEN_MAYO gene set containing 125 genes upregulated in cells exhibiting the SASP.^18^ We observed greater variance in enrichment scores at passage 3 compared to passage 1 and final passages. This variability likely reflects the varying proliferative potential of each donor. Notably, the three donors that did reach passage 10 also had the highest GSVA scores in passage 3. Since this study used bulk RNA transcriptomics, the results represent an average signal across the entire cell population, and thus, the inherent heterogeneity of the ASC can be masked. Interestingly, cells from donor B already exhibited a high enrichment score of the gene set in passage 3. It is possible that a relatively large subset of senescent ASC was already present at this stage, along with proliferating ASC. Consequently, the observed gene expression profile may reflect a continuum of cell states rather than a uniform population. Despite this limitation, senescence biomarkers must still be quantifiable at the population level to be useful in quality control testing. While single-cell RNA sequencing could elaborate on population heterogeneity, it is not feasible for routine quality control assays. Flow cytometry, however, could offer a method for accurately assessing the fraction of senescent cells within the population at single cell and protein level,^45^ and the selected biomarker candidates could be explored with this method.

Based on the GSVA analysis, we anticipated a degree of variability in the expression of individual genes related to senescence, aging, and proliferation among cultures from the five donors in passage 3. Therefore, we focused on passage 1 and final passage in the variance partition analysis, aiming to identify genes with either consistently low or high expression in ASC in early passage vs final passage, that is, genes where variance in expression was mainly attributable to passages, rather than donor. While it is true that the genes subjected to variance partitioning were selected according to a high and significant LFC between passage 1 and final passage, it remains noteworthy that numerous genes displayed transcriptional changes primarily driven by replicative exhaustion, and the regulation was consistent across donors. This strengthens the feasibility of identifying universal markers of senescence. The higher residual variance among upregulated genes may reflect underlying heterogeneity or regulatory influences not captured in the model, such as difference in cell cycle state or stochastic gene expression.

The 20 downregulated genes constitute a particularly robust and internally consistent core signal. These genes are primarily related to cell proliferation and cancer. These included several kinesins KIFC1, KIF2C, KIF14 and KIF18B,^46^^,^^47^ as well as the kinase BUB1 and its receptor BUB1B,^48^ AURKB,^49^ CDC20,^50^^,^^51^ KNL1,^52^ NUF2,^53^ SKA3,^54^ TTK^55^ all of which are involved in chromosome assembly, alignment, and segregation during mitosis. Additional downregulated genes PIMREG,^56^ DEPDC1,^57^ PKMYT,^58^ TROAP,^59^^,^^60^ UBE2C,^47^ LMN1,^61^ and TNC^62^ were overall related to proliferation or found downregulated in senescent cells. All genes display uniformly large fold changes with low donor-associated variance. This consistency suggests that loss of proliferative competence is a highly conserved feature of replicative senescence and that a limited subset of these markers may serve as reliable indicators of replicative exhaustion.

Among the 20 upregulated genes, most were related to senescence or aging in MSC or other investigated tissues or cells, these included: CRYAB, DAW1, TMEM178B, SVEP1, CACNB4,^59^ EFEMP1,^63^ GALNT5, KRT34, SYNPO2, SERPINB2,^64^ EPAS1,^65^ MFGE8, SRGN, SERPINE2,^42^ MOXD1.^66^ Upregulation of MGARP is related to mitochondrial remodeling^67^ and the Linker histone H1-2 is involved in DNA damage repair.^68^ Interestingly, MELTF was previously reported to be downregulated in aging MSC from bone marrow, suggesting that it may not serve as a robust marker of senescence.^69^

The two highly upregulated genes MEST and SLC43A2 were not individually associated with senescence or aging in the existing literature. However, the majority of the 20 up- and 20 downregulated genes, including MEST and SLC43A2, were found in a recently published cellular senescence gene signature comprising 1259 genes, identified through a meta-analysis of 20 publicly available senescence transcriptomic datasets.^70^^,^^71^ This overlap supports the relevance of our selected genes to senescence, even in cases where individual gene associations have not yet been described in detail. We conclude that the tested ASC products retain robust proliferative capacity during early passages following thawing, while extended proliferative longevity is donor dependent.

The tested ASC product maintained both PDT and morphology comparable to ASC in passage 1 for several passages, but with a progressing change in morphology and reduced ability to proliferate over later passages. We identified biologically relevant biomarker candidates, suitable for the development of a quality control assay related to senescence. We therefore state that exploration of an increase in population doublings in the production of ASC-based cell therapy is relevant. The identified 40 biomarkers are not intended to be applied as individual decision-making markers. Rather, they represent a senescence-associated discovery set, and as a next step, we suggest increasing the cumulative population doublings in the production process and investigate how this affects cellular function, such as inhibition of lymphocyte proliferation or secretion of angiogenic factors, and how the selected biomarkers correlate. Finally, neither potency assays nor a quality control assay focusing on senescence are individually sufficient but should constitute a matrix incorporating, for example, quantified gene expression, flow cytometry, and secretion of proteins.^72–74^

## Supplementary Material

szag011_Supplementary_Data

## Data Availability

The data underlying this article are available in the Gene Expression Omnibus (GEO) repository under accession number GSE309666. During the preparation of this work the author(s) used M365 Copilot to improve and correct text and GITHUB copilot to aid in R coding. After using this tool/service, the author(s) reviewed and edited the content as needed and take(s) full responsibility for the content of the publication.
