# Supplementary material for: Senescence profiling and biomarker identification in cell product based on adipose tissue–derived mesenchymal stromal cells
Source: Stem Cells Transl Med. 2026 Mar 7;15(3):szag011. doi: 10.1093/stcltm/szag011 (PMC12965767; doi:10.1093/stcltm/szag011)
Supplement: szag011_Supplementary_Data [file szag011_supplementary_data.docx]

Supplementary Information

# Title: Senescence Profiling and Biomarker Identification in Cell Product based on Adipose Tissue-derived Mesenchymal Stromal Cells

# Authors and Affiliations

Ellen Mønsted Johansen^1^, Cecilie Hoeeg^1,2^, Rebekka Harary Søndergaard^1,5^, Lisbeth Drozd Højgaard^1^, Laura Lykke Lethager^1^, Stine Bangsgaard^1^, Jens Kastrup^1,5^, Tu Hu^3^, Thomas Litman^4^, Morten Juhl Nørgaard^1^

^1^Cardiology Stem Cell Centre, The Heart Centre, University Hospital of Copenhagen Rigshospitalet, Copenhagen, Denmark
^2^Cluster for Molecular Imaging University of Copenhagen, Copenhagen, Denmark
^3^True Signal, Copenhagen, Denmark
^4^Department of Immunology and Microbiology, University Of Copenhagen, Copenhagen, Denmark
^5^CelltoCure Aps, Birkerød, Denmark

**Supplementary Figure 1** Log2 Normalized Expression Levels of the Surface Markers CD73/NT5E, CD90/THY1, and CD105/ENG across passages. Each panel compares normalized counts for donors at Passage 1 (n=5), Passage 3(n=5), Passage 6(n=2), and Final Passage(n=5). Distinct shapes and colors represent individual donors. Boxplots show mean and SD.
